# Quantitative genetic analysis of agronomic and morphological traits in sorghum, *Sorghum bicolor*

**DOI:** 10.3389/fpls.2015.00945

**Published:** 2015-11-03

**Authors:** Riyazaddin Mohammed, Ashok K. Are, Ramaiah Bhavanasi, Rajendra S. Munghate, Polavarapu B. Kavi Kishor, Hari C. Sharma

**Affiliations:** ^1^International Crops Research Institute for the Semi-Arid TropicsPatancheru, India; ^2^Department of Genetics, Osmania UniversityHyderabad, India

**Keywords:** sorghum, combining ability, heritability, agronomic traits, morphological traits, GCA, SCA, grain yield

## Abstract

The productivity in sorghum is low, owing to various biotic and abiotic constraints. Combining insect resistance with desirable agronomic and morphological traits is important to increase sorghum productivity. Therefore, it is important to understand the variability for various agronomic traits, their heritabilities and nature of gene action to develop appropriate strategies for crop improvement. Therefore, a full diallel set of 10 parents and their 90 crosses including reciprocals were evaluated in replicated trials during the 2013–14 rainy and postrainy seasons. The crosses between the parents with early- and late-flowering flowered early, indicating dominance of earliness for anthesis in the test material used. Association between the shoot fly resistance, morphological, and agronomic traits suggested complex interactions between shoot fly resistance and morphological traits. Significance of the mean sum of squares for GCA (general combining ability) and SCA (specific combining ability) of all the studied traits suggested the importance of both additive and non-additive components in inheritance of these traits. The GCA/SCA, and the predictability ratios indicated predominance of additive gene effects for majority of the traits studied. High broad-sense and narrow-sense heritability estimates were observed for most of the morphological and agronomic traits. The significance of reciprocal combining ability effects for days to 50% flowering, plant height and 100 seed weight, suggested maternal effects for inheritance of these traits. Plant height and grain yield across seasons, days to 50% flowering, inflorescence exsertion, and panicle shape in the postrainy season showed greater specific combining ability variance, indicating the predominance of non-additive type of gene action/epistatic interactions in controlling the expression of these traits. Additive gene action in the rainy season, and dominance in the postrainy season for days to 50% flowering and plant height suggested G X E interactions for these traits.

## Introduction

Sorghum [*Sorghum bicolor* (L.) Moench] is an important crop grown primarily in warm and dry climates with a wide range of adaptability to various agro-ecological conditions. It is the fifth most important food crop after wheat, rice, maize, and barley (FAO, 2004), and is widely grown in the semi-arid regions. It is the staple food for 600 million people living in the semi-arid regions. India is the third largest sorghum producer after Nigeria and United States of America, with 6.25 million hectares of area under sorghum cultivation, and with a total production of 5.98 million tonnes (FAOSTAT, 2012).

Information on inheritance of agronomic and morphological traits is useful for improving genotypic performance across environments. In sorghum, both the additive and non-additive type of gene action governs the inheritance of morphological and agronomic traits (Nimbalkar and Bapat, 1992; Umakanth et al., 2002; Mohammed Maarouf, 2009) with considerable amount of G X E interaction (Jayanthi et al., 1996; Dhillon et al., 2006; Aruna et al., 2011a).

Most of the morphological traits in sorghum are associated with one or more economically important traits, and will be helpful in selecting the high yielding sorghum genotypes. Brown midrib increases the fodder quality, while the presence of awns acts as a mechanical barrier to bird damage (Porter et al., 1978; Kullaiswamy and Goud, 1983). Genotypes with tan-colored plants showed resistance to various fungal diseases while the genotypes with closed glumes are resistant to grain mold (Melake-Berhan et al., 1996; Murty, 2000).

Although, considerable progress has been made in identifying insect-resistant sorghums (Sharma, 1993; Sharma et al., 2003), but there is little progress in developing insect-resistant high yielding varieties for cultivation by the farmers. This is largely because of the lack of knowledge on inheritance of the agronomic and morphological characteristics associated with insect resistance and grain yield (Sharma et al., 2005; Riyazaddin et al., 2015). The combining ability analysis is useful to understand the nature of gene action, and has been used by the breeders to select the suitable parents for the crossing program. An understanding of the inheritance of morphological and agronomic traits will be helpful in combining the genes for insect resistance and desirable agronomic traits and grain characteristics to increase production and productivity of sorghum. Therefore, we developed a full diallel involving 10 parents to study the inheritance of morphological and agronomic traits. The combining ability studies will be helpful to identify genotypes which can be utilized in the hybridization.

## Materials and methods

### Experimental material

Based on *per se* performance of sorghum genotypes in the field against shoot fly, *Atherigona soccata* and molecular diversity, 10 morphologically and genetically diverse sorghum genotypes (Annexure I in Supplementary Material) adapted to the rainy and postrainy seasons were selected and crossed in all possible combinations, which generated 45 direct crosses and 45 reciprocal crosses. These crosses along with the parents were evaluated in a randomized complete block design in three replications during the 2013–14 rainy and postrainy seasons at the International Crops Research Institute for the Semi-Arid Tropics (ICRISAT), Patancheru, Hyderabad, Telangana, India (latitude 17.53°N, longitude 78.27°E, and altitude of 545 m).

Sowing of the test genotypes was carried out using a two cone planter. Each test plot consists of a row length of 2.0 m and a row to row spacing of 75 cm. A distance of 10 cm was maintained in-between the plants within a row. Application of a basal dose of Ammonium phosphate to the field was carried out at 100 kg/ha. Each parent was sown in two rows, and a single row of F_1_. Thinning of the test plots was carried out at 7 days after seedling emergence (DAE) and a plant population of 40 plants were retained in a test plot. At 30 DAE earthing up was carried out along with top dressing with urea at 100 kg/ha. During the postrainy season furrow irrigation was given to the experimental material. One set of the replicated test material was grown under protected conditions (application of carbofuran 3G granules in the leaf whorls at 7 days after seedling emergence, and cypermethrin spray after 5 days) to record the agronomic and morphological traits in the undamaged plants during the rainy and postrainy seasons.

### Observations

#### Agronomic characteristics

Data were recorded on days to 50% flowering, plant height, agronomic score, 100 seed weight, and grain yield. Days to 50% flowering was recorded when half of the panicles and nearly 50% of plants in the plot had attained the anthesis stage. Height of three plants was measured from the base of the plant to the tip of the panicle at physiological maturity in plants selected at random within a test plot. The agronomic desirability of the genotype was recorded at crop maturity on a 1–5 scale (1 = high productive potential, and 5 = poor productive potential). Data on 100 seed weight and grain yield/plot for parents, and grain yield/5 plants for F_1_s were recorded after crop harvest.

#### Morphological characteristics

Inflorescence exsertion was scored on a 1–4 scale (1 = panicle fully exserted, and 4 = panicle recurved); panicle compactness on a 1–3 scale (1 = loose panicle, and 3 = compact panicle); panicle shape on a 1–4 scale (1 = erect panicle, and 4 = elliptic panicle); glume coverage on a 1–9 scale (1 = 25% grain covered with glumes, and 9 = glumes longer than the grain); awns on 1–2 scale (1 = absence of awns, 2 = presence of awns); grain luster on 1–2 scale (1 = non-lustrous grain, 2 = lustrous grain); and grain color on a 1–5 scale (1 = white colored grain and 5 = buff colored grain) (IBPGR and ICRISAT, 1993).

### Statistical analysis

Analysis of variance (ANOVA) was carried out using GenStat® 13th version (GenStat, 2010). *F*-test was used to test the significance differences between the genotypes, and least significance difference (LSD) for comparing the genotypic means at *P* ≤ 0.05. Simple correlation coefficients were calculated to determine the association between the traits studied. Partitioning of the combining abilities (GCA and SCA) was done using the method 1 and model 1 of Griffing (1956), that provides the information on nature of parents, and the hybrid performance, using Windowstat (Indostat Services, 2004) software. The coefficient of variations at phenotypic and genotypic level variation was estimated using the formula adopted by Johnson et al. (1955) and predictability ratio using Baker (1978).

## Results

### Agronomic traits

Evaluation of 10 parents and 90 F_1_s, including the reciprocals, showed significant differences for all the traits studied across seasons at *P* ≤ 0.01. Days to 50% flowering was ranged from 61 to 81 days in the rainy season, 56–78 days in the postrainy season (Table 1). Almost all the crosses flowered early, with few exceptions. Crosses between the parents with early- and late-flowering were early-flowering, indicating dominance of earliness for anthesis in the test material used. The crosses CSV 15 X ICSV 25019, CSV 15 X PS 35805, ICSV 25019 X CSV 15, ICSV 25019 X PS 35805, ICSV 25019 X IS 2123, ICSV 25019 X Swarna, PS 35805 X CSV 15, PS 35805 X ICSV 25019, PS 35805 X IS 2146, PS 35805 X Swarna, Swarna X ICSV 25019, and Swarna X PS 35805 exhibited moderate plant height across seasons. Parents and the crosses with moderate plant height can be exploited in developing the commercial hybrids amenable for mechanical harvesting.

**Table 1:** **Agronomic characteristics of sorghum genotypes (Parents and F_1_'s) evaluated for resistance to sorghum shoot fly, *A. soccata* across seasons (ICRISAT, Patancheru, 2013–14)**.

**Pedigree**	**Days to 50% flowering**		**Plant height (cm)**		**100 seed weight (g)**		**Grain yield (t/ha)**		**Agronomic score**	
	**2013 R**	**2013 PR**	**2013 R**	**2013 PR**	**2013 R**	**2013 PR**	**2013 R**	**2013 PR**	**2013 R**	**2013 PR**
**PARENTS**										
ICSV 700	81	77	309	189	2.30	2.37	0.79	5.02	5.00	3.67
Phule Anuradha	62	63	259	179	2.90	4.23	1.22	6.51	4.67	4.33
M 35-1	75	72	306	187	2.40	3.50	0.66	6.87	5.00	3.67
CSV 15	71	61	254	180	2.50	3.03	1.54	5.23	3.00	3.00
ICSV 25019	65	64	131	109	2.30	2.10	1.90	3.01	2.00	3.00
PS 35805	69	65	121	102	2.20	2.37	1.70	3.12	2.00	3.00
IS 2123	73	72	283	176	2.13	2.50	0.77	5.64	5.00	4.33
IS 2146	68	73	279	181	1.80	2.33	0.88	5.56	5.00	4.67
IS 18551	78	78	313	203	1.70	2.23	0.51	4.23	5.00	3.33
Swarna	67	63	166	138	3.30	3.77	1.89	5.17	2.00	2.00
**DIRECT CROSSES**										
ICSV 700 X Phule Anuradha	65	75	314	236	2.73	3.77	3.58	15.18	4.33	2.67
ICSV 700 X M 35-1	65	70	321	218	2.63	4.00	3.73	12.51	3.67	3.33
ICSV 700 X CSV 15	75	71	327	209	2.37	3.30	4.73	14.18	2.33	4.00
ICSV 700 X ICSV 25019	67	68	308	199	2.80	3.63	4.80	12.72	3.00	3.00
ICSV 700 X PS 35805	67	67	309	204	2.47	3.07	6.25	12.13	3.33	3.67
ICSV 700 X IS 2123	68	69	303	219	2.50	3.40	4.12	13.73	4.67	4.33
ICSV 700 X IS 2146	69	71	318	208	2.53	3.53	2.57	5.49	4.00	3.67
ICSV 700 X IS 18551	67	70	339	231	2.40	3.30	5.31	11.87	3.67	3.33
ICSV 700 X Swarna	67	68	319	213	3.07	4.03	4.10	10.46	5.00	3.00
Phule Anuradha X M 35-1	64	66	273	202	2.60	4.37	2.93	11.99	4.00	3.33
Phule Anuradha X CSV 15	62	59	302	202	3.00	4.07	1.97	8.94	4.00	3.33
Phule Anuradha X ICSV 25019	61	60	280	183	2.87	3.97	2.58	10.69	3.67	2.67
Phule Anuradha X PS 35805	62	61	280	177	2.97	3.53	5.89	14.08	3.33	3.33
Phule Anuradha X IS 2123	63	64	280	188	2.60	3.83	3.40	12.40	4.33	4.00
Phule Anuradha X IS 2146	64	66	292	187	2.20	4.00	2.89	7.17	4.00	3.67
Phule Anuradha X IS 18551	65	65	310	208	2.27	3.47	4.68	12.70	3.33	4.00
Phule Anuradha X Swarna	63	62	297	199	3.43	4.33	4.76	9.44	3.33	3.33
M 35-1 X CSV 15	64	70	317	211	2.97	3.37	4.47	11.58	3.67	3.00
M 35-1 X ICSV 25019	63	63	304	203	2.77	3.83	3.34	13.94	3.33	3.33
M 35-1 X PS 35805	63	64	296	171	2.73	3.43	4.00	11.51	2.67	3.00
M 35-1 X IS 2123	66	70	296	198	2.57	3.50	3.84	14.34	4.67	4.33
M 35-1 X IS 2146	64	69	293	198	2.20	3.93	1.96	7.17	4.67	4.00
M 35-1 X IS 18551	67	70	322	211	2.37	3.17	4.69	14.47	3.67	3.00
M 35-1 X Swarna	69	67	314	208	2.37	3.13	4.49	11.56	3.33	3.33
CSV 15 X ICSV 25019	63	56	229	149	2.47	3.50	6.94	13.28	1.67	2.33
CSV 15 X PS 35805	65	57	250	158	2.50	3.33	7.16	12.62	2.00	2.67
CSV 15 X IS 2123	66	61	298	184	2.67	3.23	7.95	14.49	4.33	3.67
CSV 15 X IS 2146	64	63	290	197	2.77	3.30	2.88	7.34	3.33	3.67
CSV 15 X IS 18551	65	63	340	214	2.47	2.97	5.72	13.77	3.00	3.00
CSV 15 X Swarna	65	59	308	198	3.10	3.67	4.38	11.11	3.67	2.33
ICSV 25019 X PS 35805	66	60	127	104	1.97	2.43	4.33	7.57	2.00	3.00
ICSV 25019 X IS 2123	64	63	258	167	2.87	3.50	4.95	13.61	4.67	3.33
ICSV 25019 X IS 2146	63	64	273	173	2.80	3.70	2.41	6.99	4.33	3.67
ICSV 25019 X IS 18551	65	65	302	209	2.37	2.90	4.18	12.91	3.33	2.67
ICSV 25019 X Swarna	61	62	187	140	3.27	3.00	7.65	10.16	1.67	2.67
PS 35805 X IS 2123	67	63	276	161	2.73	2.90	3.88	12.48	4.33	3.67
PS 35805 X IS 2146	63	61	261	180	2.53	3.20	2.14	7.20	3.67	3.33
PS 35805 X IS 18551	65	63	318	202	2.43	2.60	4.11	11.42	3.00	3.00
PS 35805 X Swarna	64	61	184	139	2.73	2.80	5.36	10.40	2.00	2.67
IS 2123 X IS 2146	68	71	292	196	1.97	3.10	2.01	6.51	4.67	4.33
IS 2123 X IS 18551	69	73	298	207	2.00	2.50	2.49	10.32	5.00	4.33
IS 2123 X Swarna	67	67	290	198	3.20	3.60	5.07	10.86	4.33	4.33
IS 2146 X IS 18551	67	72	290	211	2.10	3.03	2.23	8.33	4.67	4.67
IS 2146 X Swarna	63	62	297	210	2.63	3.87	3.13	5.40	4.33	3.67
IS 18551 X Swarna	68	65	327	222	2.90	3.50	5.06	12.10	3.67	3.00
**RECIPROCAL CROSSES**										
Phule Anuradha X ICSV 700	66	64	316	213	2.67	4.17	3.93	10.04	3.67	3.67
M 35-1 X ICSV 700	68	69	317	219	2.67	4.30	6.54	11.72	4.00	3.67
M 35-1 X Phule Anuradha	65	64	283	212	2.57	4.10	3.25	10.90	3.33	4.33
CSV 15 X ICSV 700	69	68	333	206	2.47	3.27	5.82	15.09	2.33	3.00
CSV 15 X Phule Anuradha	64	59	310	218	3.10	4.10	4.68	11.48	3.33	3.67
CSV 15 X M 35-1	63	64	302	212	2.90	4.13	4.14	14.25	3.67	3.67
ICSV 25019 X ICSV 700	68	67	311	198	2.57	3.47	5.02	12.99	3.00	3.00
ICSV 25019 X Phule Anuradha	61	59	282	187	3.30	4.13	5.97	11.13	3.00	2.67
ICSV 25019 X M 35-1	66	69	308	186	2.90	3.50	5.47	15.24	3.67	3.33
ICSV 25019 X CSV 15	64	59	230	158	2.47	3.00	7.46	11.77	2.00	2.67
PS 35805 X ICSV 700	71	66	309	203	2.53	3.07	6.29	12.33	2.67	3.33
PS 35805 X Phule Anuradha	63	68	288	211	2.93	3.27	4.20	17.28	3.33	3.33
PS 35805 X M 35-1	65	64	298	197	2.77	3.53	4.52	14.00	4.67	2.67
PS 35805 X CSV 15	65	57	238	152	2.40	2.90	6.74	10.79	2.83	3.00
PS 35805 X ICSV 25019	67	61	127	104	2.00	2.07	3.10	7.02	2.00	3.00
IS 2123 X ICSV 700	68	70	312	208	2.57	3.47	4.76	12.21	5.00	4.33
IS 2123 X Phule Anuradha	65	67	288	192	2.57	3.63	3.89	11.64	5.00	3.33
IS 2123 X M 35-1	69	68	312	197	2.37	3.50	2.90	13.93	4.33	4.33
IS 2123 X CSV 15	65	64	290	196	2.83	3.20	7.04	13.29	5.00	3.67
IS 2123 X ICSV 25019	67	64	269	161	2.77	3.20	5.09	13.91	4.33	3.67
IS 2123 X PS 35805	67	59	271	171	2.90	3.13	4.60	12.58	4.33	3.67
IS 2146 X ICSV 700	67	71	320	209	2.57	3.17	2.21	5.45	4.33	4.33
IS 2146 X Phule Anuradha	62	64	288	191	2.43	3.73	2.40	6.72	4.67	4.33
IS 2146 X M 35-1	66	68	293	199	2.20	3.70	2.76	7.08	5.00	4.67
IS 2146 X CSV 15	64	57	307	188	2.73	3.37	3.21	6.90	4.33	3.67
IS 2146 X ICSV 25019	64	64	270	166	2.37	3.43	1.96	6.93	4.50	4.67
IS 2146 X PS 35805	64	63	280	169	2.40	3.33	2.10	7.38	4.00	4.33
IS 2146 X IS 2123	68	69	300	199	1.90	3.30	2.32	6.62	4.67	4.67
IS 18551 X ICSV 700	71	71	343	211	2.13	3.20	4.82	10.72	4.00	3.33
IS 18551 X Phule Anuradha	65	65	308	209	2.30	3.43	4.84	14.73	3.67	3.67
IS 18551 X M 35-1	72	70	320	221	2.30	3.23	5.95	13.67	3.33	3.67
IS 18551 X CSV 15	67	64	336	228	2.47	2.97	6.16	12.98	3.33	3.00
IS 18551 X ICSV 25019	66	63	306	198	2.37	2.97	3.12	13.75	3.00	3.00
IS 18551 X PS 35805	69	64	308	207	2.23	2.77	4.28	13.02	3.00	3.00
IS 18551 X IS 2123	73	69	293	208	2.23	2.83	4.00	11.36	5.00	4.33
IS 18551 X IS 2146	68	71	320	197	1.87	2.93	2.35	5.85	5.00	4.33
Swarna X ICSV 700	68	66	318	226	2.90	4.20	5.82	8.62	3.33	3.00
Swarna X Phule Anuradha	61	58	313	204	3.30	4.60	4.73	9.44	3.67	3.33
Swarna X M 35-1	64	62	316	193	3.13	4.47	4.36	10.39	3.33	3.00
Swarna X CSV 15	63	57	299	217	2.77	4.10	4.92	11.58	3.67	2.67
Swarna X ICSV 25019	61	59	187	142	2.83	2.90	7.38	9.46	2.00	2.67
Swarna X PS 35805	65	62	191	149	2.57	3.03	8.70	9.10	1.67	2.33
Swarna X IS 2123	64	63	294	177	2.90	3.73	4.94	10.79	4.33	4.00
Swarna X IS 2146	63	60	287	211	2.60	3.50	2.36	5.92	3.33	3.33
Swarna X IS 18551	65	67	330	230	2.83	3.40	5.27	11.69	3.67	3.00
Mean	66	65	286	191	2.60	3.38	4.10	10.43	3.69	3.46
SE ±	1.13	1.40	6.21	5.83	0.12	0.15	0.71	1.04	0.35	0.40
Vr (99, 198)	10.09^**^	10.90^**^	56.80^**^	22.91^**^	8.66^**^	12.87^**^	6.37^**^	9.65^**^	7.48^**^	2.41^**^
LSD (P 0.05)	3.14	3.90	17.33	16.26	0.34	0.42	1.97	2.90	0.97	1.12

** *F probability significant at P 0.01; R, rainy season; PR, postrainy season*.

Ten crosses exhibited high 100 seed weight and grain yield with good agronomic desirability. Grain yield of the crosses CSV 15 X IS 2123, ICSV 25019 X Swarna, PS 35805 X Swarna, IS 2123 X CSV 15, IS 2123 X ICSV 25019, IS 2123 X Swarna, Swarna X ICSV 25019, and Swarna X IS 2123 was high in the rainy season.

### Morphological and grain characteristics

All the panicle traits showed significant variability among the genotypes for all the characteristics studied, in both the rainy and postrainy seasons with significant variance ratio (*P* ≤ 0.01) (Table 2). The mean scores for inflorescence exsertion were 1.90 and 2.41; for panicle compactness 2.30 and 2.63; and for glume coverage 2.00 and 1.71, respectively, in the rainy and postrainy seasons.

**Table 2:** **Panicle and grain characteristics of sorghum genotypes (Parents and F_1_'s) evaluated for resistance to sorghum shoot fly, *A. soccata* across seasons (ICRISAT, Patancheru, 2013–14)**.

**Pedigree**	**Inflorescence exsertion**		**Panicle compactness**		**Panicle shape**	**Glume coverage**		**Awns**		**Grain luster**
	**2013 R**	**2013 PR**	**2013 R**	**2013 PR**	**2013 PR**	**2013 R**	**2013 PR**	**2013 R**	**2013 PR**	**2013 PR**
**PARENTS**										
ICSV 700	2.00	2.00	2.00	3.00	4.00	5.00	5.00	2.00	2.00	2.00
Phule Anuradha	2.33	3.00	2.00	3.00	4.00	3.00	1.00	2.00	2.00	2.00
M 35-1	2.00	2.00	2.00	3.00	4.00	3.00	1.00	2.00	2.00	2.00
CSV 15	2.00	1.00	2.00	2.00	2.00	1.00	1.00	1.00	1.00	2.00
ICSV 25019	2.67	2.00	3.00	3.00	4.00	1.00	1.00	1.00	1.00	2.00
PS 35805	3.00	2.00	3.00	3.00	4.00	1.00	1.00	1.00	1.00	2.00
IS 2123	2.33	4.00	3.00	3.00	3.00	3.00	1.00	2.00	2.00	2.00
IS 2146	2.00	4.00	3.00	3.00	3.00	3.00	1.00	2.00	2.00	2.00
IS 18551	2.00	2.00	2.00	3.00	4.00	9.00	9.00	2.00	2.00	1.00
Swarna	1.00	1.00	2.00	2.00	1.00	1.00	1.00	1.00	1.00	2.00
**DIRECT CROSSES**										
ICSV 700 x Phule Anuradha	2.00	2.67	2.00	3.00	4.00	1.67	1.00	2.00	2.00	2.00
ICSV 700 x M 35-1	2.00	2.33	2.00	3.00	4.00	1.67	1.00	2.00	2.00	2.00
ICSV 700 x CSV 15	1.67	1.33	2.00	2.00	1.33	1.00	1.00	1.00	1.00	2.00
ICSV 700 x ICSV 25019	2.00	1.67	2.00	2.33	2.00	1.00	1.00	1.00	1.00	2.00
ICSV 700 x PS 35805	2.00	1.00	2.00	3.00	4.00	1.67	1.00	1.00	1.00	2.00
ICSV 700 x IS 2123	2.00	4.00	3.00	3.00	3.00	1.67	1.00	2.00	2.00	2.00
ICSV 700 x IS 2146	2.00	3.00	2.67	3.00	3.33	2.33	1.00	2.00	2.00	2.00
ICSV 700 x IS 18551	1.33	2.33	2.00	3.00	4.00	5.67	4.33	2.00	2.00	1.67
ICSV 700 x Swarna	2.00	1.00	2.00	2.00	1.00	1.67	1.67	1.00	1.00	2.00
Phule Anuradha x M 35-1	2.00	3.00	2.00	2.67	3.00	1.00	1.00	2.00	2.00	2.00
Phule Anuradha x CSV 15	1.67	1.67	2.00	2.00	1.33	1.00	1.00	1.00	1.00	2.00
Phule Anuradha x ICSV 25019	2.00	2.33	2.00	2.00	1.00	1.00	1.00	1.00	1.00	2.00
Phule Anuradha x PS 35805	2.00	3.00	2.33	2.33	2.00	1.00	1.00	1.00	1.00	2.00
Phule Anuradha x IS 2123	2.67	4.00	3.00	3.00	3.00	1.67	1.00	2.00	2.00	2.00
Phule Anuradha x IS 2146	2.67	4.00	2.67	3.00	3.00	1.67	1.00	2.00	2.00	2.00
Phule Anuradha x IS 18551	2.00	2.33	2.00	3.00	4.00	3.00	5.00	2.00	2.00	2.00
Phule Anuradha x Swarna	1.67	1.33	2.00	2.00	1.00	1.00	1.00	1.00	1.00	2.00
M 35-1 x CSV 15	1.00	1.00	2.00	2.33	2.67	1.00	1.67	1.00	1.00	2.00
M 35-1 x ICSV 25019	1.00	1.00	2.00	2.00	1.00	1.00	1.00	1.00	1.00	2.00
M 35-1 x PS 35805	1.00	1.67	2.33	3.00	4.00	2.33	1.00	1.00	1.00	2.00
M 35-1 x IS 2123	2.00	4.00	2.67	3.00	3.00	1.67	1.00	2.00	2.00	2.00
M 35-1 x IS 2146	2.33	4.00	2.67	3.00	3.00	1.00	1.00	2.00	2.00	2.00
M 35-1 x IS 18551	1.67	2.33	2.00	3.00	4.00	5.00	4.33	2.00	2.00	2.00
M 35-1 x Swarna	2.00	1.33	2.00	3.00	4.00	3.00	1.00	1.00	1.00	2.00
CSV 15 x ICSV 25019	2.33	3.00	2.00	2.00	1.33	1.00	1.00	1.00	1.00	2.00
CSV 15 x PS 35805	2.33	1.67	2.00	2.00	1.33	1.00	1.00	1.00	1.00	2.00
CSV 15 x IS 2123	2.00	4.00	3.00	3.00	3.33	1.67	1.00	1.00	1.00	2.00
CSV 15 x IS 2146	2.00	2.33	2.67	3.00	3.67	1.00	1.00	1.00	1.00	2.00
CSV 15 x IS 18551	1.33	1.33	2.00	2.00	1.00	4.33	3.67	1.33	1.00	2.00
CSV 15 x Swarna	1.00	1.00	2.00	1.00	2.00	1.67	1.00	1.00	1.00	2.00
ICSV 25019 x PS 35805	3.00	3.00	2.33	3.00	4.00	1.00	1.00	1.00	1.00	2.00
ICSV 25019 x IS 2123	3.00	4.00	3.00	3.00	3.00	1.00	1.00	1.00	1.00	2.00
ICSV 25019 x IS 2146	2.33	4.00	2.67	3.00	3.67	1.00	1.00	1.00	1.00	2.00
ICSV 25019 x IS 18551	1.00	1.00	2.00	2.67	3.00	3.00	3.00	1.00	1.00	2.00
ICSV 25019 x Swarna	1.00	1.33	2.00	2.00	1.00	1.00	1.00	1.00	1.00	2.00
PS 35805 x IS 2123	2.33	4.00	3.00	3.00	3.00	2.33	1.00	1.00	1.00	2.00
PS 35805 x IS 2146	2.33	3.33	3.00	3.00	3.67	1.00	1.00	1.00	1.00	2.00
PS 35805 x IS 18551	1.33	1.67	2.00	2.00	1.00	3.67	3.67	1.00	1.00	1.67
PS 35805 x Swarna	1.00	1.00	2.00	2.00	1.00	1.00	1.00	1.00	1.00	2.00
IS 2123 x IS 2146	3.00	4.00	3.00	3.00	3.00	1.67	1.00	2.00	2.00	2.00
IS 2123 x IS 18551	2.67	4.00	3.00	3.00	3.00	4.33	4.33	2.00	2.00	2.00
IS 2123 x Swarna	2.00	4.00	2.67	3.00	3.67	1.00	1.00	1.00	1.00	2.00
IS 2146 x IS 18551	2.33	3.00	3.00	3.00	3.00	2.33	3.67	2.00	2.00	2.00
IS 2146 x Swarna	1.00	2.00	2.00	3.00	4.00	1.67	1.00	1.00	1.00	2.00
IS 18551 x Swarna	1.00	1.00	2.00	2.00	1.00	4.33	4.33	1.00	1.00	2.00
**RECIPROCAL CROSSES**										
Phule Anuradha x ICSV 700	2.00	3.00	2.00	3.00	4.00	1.67	1.00	2.00	2.00	2.00
M 35-1 x ICSV 700	1.67	2.67	2.00	3.00	4.00	1.67	1.00	2.00	2.00	2.00
M 35-1 x Phule Anuradha	2.33	2.00	2.00	2.33	2.00	1.00	1.00	2.00	1.67	1.67
CSV 15 x ICSV 700	1.67	1.33	2.00	2.33	2.33	1.67	1.00	1.00	1.00	2.00
CSV 15 x Phule Anuradha	1.33	1.33	2.00	2.00	1.67	1.00	1.00	1.00	1.00	2.00
CSV 15 x M 35-1	1.33	1.33	2.00	2.00	1.33	1.00	1.67	1.00	1.00	2.00
ICSV 25019 x ICSV 700	1.00	1.67	2.00	2.67	3.00	1.67	1.00	1.00	1.00	2.00
ICSV 25019 x Phule Anuradha	1.33	1.33	2.00	2.33	2.00	1.67	1.00	1.00	1.00	2.00
ICSV 25019 x M 35-1	1.33	1.67	2.00	2.67	3.00	1.67	1.00	1.00	1.00	2.00
ICSV 25019 x CSV 15	2.00	2.67	2.00	2.00	1.67	1.00	1.00	1.00	1.00	2.00
PS 35805 x ICSV 700	1.67	1.33	2.00	3.00	4.00	1.00	1.00	1.00	1.00	2.00
PS 35805 x Phule Anuradha	1.67	2.00	2.00	3.00	4.00	1.00	1.00	1.00	1.00	2.00
PS 35805 x M 35-1	1.00	1.33	2.00	3.00	4.00	1.67	1.00	1.00	1.00	2.00
PS 35805 x CSV 15	2.33	2.33	2.00	2.00	1.67	1.00	1.00	1.00	1.00	2.00
PS 35805 x ICSV 25019	3.00	3.00	2.00	3.00	4.00	1.00	1.00	1.00	1.00	2.00
IS 2123 x ICSV 700	2.67	4.00	3.00	3.00	3.00	2.33	1.00	2.00	2.00	2.00
IS 2123 x Phule Anuradha	2.67	4.00	3.00	3.00	3.00	1.67	1.00	2.00	2.00	2.00
IS 2123 x M 35-1	3.00	4.00	3.00	3.00	3.00	1.67	1.00	2.00	2.00	2.00
IS 2123 x CSV 15	2.67	4.00	2.67	3.00	3.00	1.67	1.00	1.00	1.00	2.00
IS 2123 x ICSV 25019	2.00	3.67	3.00	3.00	3.00	1.67	1.00	1.00	1.00	2.00
IS 2123 x PS 35805	2.67	3.67	3.00	3.00	3.00	1.00	1.00	1.00	1.00	2.00
IS 2146 x ICSV 700	2.33	3.67	2.67	3.00	3.33	1.67	1.00	2.00	2.00	2.00
IS 2146 x Phule Anuradha	2.67	4.00	3.00	3.00	3.00	1.67	1.00	2.00	1.67	2.00
IS 2146 x M 35-1	2.67	4.00	3.00	3.00	3.00	1.00	1.00	2.00	2.00	2.00
IS 2146 x CSV 15	1.00	3.33	2.33	3.00	4.00	1.00	1.67	1.00	1.00	2.00
IS 2146 x ICSV 25019	2.33	3.67	3.00	3.00	3.67	1.00	1.00	1.00	1.00	2.00
IS 2146 x PS 35805	2.67	3.67	3.00	3.00	3.33	1.00	1.00	1.00	1.00	2.00
IS 2146 x IS 2123	3.00	4.00	3.00	3.00	3.00	1.67	1.00	2.00	2.00	2.00
IS 18551 x ICSV 700	2.00	2.67	2.00	3.00	4.00	5.67	3.67	2.00	2.00	2.00
IS 18551 x Phule Anuradha	2.33	2.67	2.00	3.00	4.00	4.33	5.00	2.00	2.00	2.00
IS 18551 x M 35-1	2.00	2.33	2.00	3.00	4.00	5.00	5.00	2.00	2.00	2.00
IS 18551 x CSV 15	1.00	1.00	2.00	2.00	1.33	2.33	4.33	1.00	1.00	1.67
IS 18551 x ICSV 25019	1.33	1.00	2.00	2.00	1.00	3.67	3.00	1.00	1.00	2.00
IS 18551 x PS 35805	1.67	1.33	2.00	2.33	2.00	3.67	3.67	1.00	1.00	2.00
IS 18551 x IS 2123	3.00	4.00	3.00	3.00	3.00	4.33	4.33	2.00	2.00	2.00
IS 18551 x IS 2146	3.00	2.33	3.00	3.00	3.00	3.67	3.67	2.00	2.00	2.00
Swarna x ICSV 700	1.33	1.00	2.00	2.00	1.00	1.67	1.00	1.00	1.00	2.00
Swarna x Phule Anuradha	1.00	1.00	2.00	2.00	1.33	1.67	1.00	1.00	1.00	2.00
Swarna x M 35-1	1.33	1.00	2.00	2.33	1.00	1.00	1.00	1.00	1.00	2.00
Swarna x CSV 15	1.00	1.00	2.00	1.00	2.00	1.00	1.00	1.00	1.00	2.00
Swarna x ICSV 25019	1.00	1.00	2.00	2.00	1.00	1.00	1.00	1.00	1.00	2.00
Swarna x PS 35805	1.00	1.00	2.00	2.00	1.00	1.00	1.00	1.00	1.00	2.00
Swarna x IS 2123	1.67	3.00	3.00	3.00	3.33	1.00	1.00	1.00	1.00	2.00
Swarna x IS 2146	1.67	1.00	2.00	3.00	4.00	1.67	1.00	1.00	1.00	2.00
Swarna x IS 18551	1.00	1.00	2.00	2.00	1.00	4.33	5.00	1.00	1.00	2.00
Mean	1.90	2.41	2.30	2.63	2.75	2.00	1.71	1.40	1.35	1.98
SE ±	0.25	0.29	0.12	0.12	0.35	0.43	0.33	0.03	0.05	0.07
Vr	6.49^**^	15.17^**^	13.65^**^	18.62^**^	9.81^**^	10.81^**^	19.91^**^	208.27^**^	102.88^**^	3.18^**^
LSD (P 0.05)	0.69	0.81	0.33	0.32	0.99	1.20	0.92	0.09	0.13	0.19

** *F probability significant at P 0.05; R, rainy season; PR, postrainy season*.

### Association of the agronomic and morphological traits with shoot fly resistance

Days to 50% flowering, inflorescence exsertion, panicle compactness, glume coverage, and presence of awns were significantly and negatively correlated with shoot fly damage parameters across seasons, with few exceptions (Table 3). Plant height, 100 seed weight, and grain yield showed positive correlation with shoot fly damage across seasons.

**Table 3:** **Association of agronomic and panicle traits with expression of resistance to sorghum shoot fly, *Atherigona soccata* (ICRISAT, Patancheru, 2013–14)**.

**Traits**	**Plants with shoot fly eggs (%)**	**Number of shoot fly eggs/plant**	**Shoot fly deadhearts (%)**	**ORS**
Days to 50% flowering	−0.20^*^ (−0.41^**^)	0.13 (−0.07)	−0.31^**^ (−0.47^**^)	0.09 (−0.24^**^)
Plant height	0.13 (−0.01)	0.15 (−0.06)	0.35^**^ (0.07)	−0.07 (−0.02)
100 seed weight	0.21^*^ (0.28^**^)	−0.03 (−0.04)	0.39^**^ (0.32^**^)	0.13 (0.15)
Grain yield	0.16^*^ (0.03)	−0.01 (−0.17^*^)	0.24^**^ (0.05)	0.05 (0.22^*^)
Agronomic score	−0.03 (−0.43^**^)	0.09 (−0.17^*^)	0.02 (−0.41^**^)	−0.10 (−0.38^**^)
Inflorescence exsertion	−0.19^*^ (−0.37^**^)	−0.06 (−0.14)	−0.37^**^ (−0.41^**^)	−0.18^*^ (−0.31^**^)
Panicle compactness	−0.13 (−0.55^**^)	−0.01 (−0.18^*^)	−0.30^**^ (−0.60^**^)	−0.28^**^ (−0.45^**^)
Panicle shape	(−0.48^**^)	(−0.10)	(−0.49^**^)	(−0.38^**^)
Glume coverage	−0.17^*^ (−0.16^*^)	0.15 (0.08)	−0.18^*^ (−0.44^**^)	−0.01 (−0.13)
Awns	−0.12 (−0.41^**^)	0.01 (−0.07)	−0.18^*^ (−0.44^**^)	0.10 (−0.17^*^)

*, ** *Correlation coefficients significant at P 0.05 and P 0.01, respectively; ORS, overall resistance score; The values inside the parentheses are for postrainy season, whereas the values outside the parentheses are for rainy season*.

### Association between the agronomic traits

Agronomic score was positively correlated with days to 50% flowering and plant height, but negatively correlated with grain yield (Table 4). Days to 50% flowering were significantly and positively correlated with plant height, and negatively correlated with 100 seed weight and grain yield. Grain yield was positively correlated with plant height in the postrainy season, and 100 seed weight across seasons. Significant positive correlation was observed between plant height and 100 seed weight in the postrainy season.

**Table 4:** **Association between the agronomic traits in the postrainy season adapted sorghums (ICRISAT, Patancheru, 2013–14)**.

**Traits**	**Agronomic score**	**Days to 50% flowering**	**Plant height**	**100 seed weight**
Days to 50% flowering	0.24^**^ (0.45^**^)	1.00		
Plant height	0.52^**^ (0.24^**^)	0.22^*^ (0.41^**^)	1.00	
100 seed weight	−0.17 (0.01)	−0.51^**^ (−0.23^**^)	0.04 (0.43^**^)	1.00
Grain yield	−0.47^**^ (−0.21^*^)	−0.26^**^ (−0.08)	0.00 (0.36^**^)	0.36^**^ (0.21^*^)

*, ** *Correlation coefficients significant at P 0.05 and P 0.01, respectively; The values inside the parentheses are for postrainy season, whereas the values outside the parentheses are for rainy season*.

### Association between the morphological traits

Significant positive correlation was observed between inflorescence exsertion and panicle compactness, and between awns and the panicle traits across seasons (Table 5). Panicle shape was positively correlated with inflorescence exsertion, and panicle compactness in the postrainy season.

**Table 5:** **Association between the panicle traits in the postrainy season sorghums (ICRISAT, Patancheru, 2013–14)**.

**Traits**	**Inflorescence exsertion**	**Panicle compactness**	**Panicle shape**	**Glume coverage**	**Awns**
Panicle compactness	0.66^**^ (0.66^**^)	1.00			
Panicle shape	(0.46^**^)	(0.86^**^)	1.00		
Glume coverage	−0.03 (−0.14)	−0.12 (0.05)	(0.07)	1.00	
Awns	0.45^**^ (0.53^**^)	0.30^**^ (0.52^**^)	(0.44^**^)	0.44^**^ (0.29^**^)	1.00
Grain luster	(0.10)	(0.01)	(−0.03)	(−0.55^**^)	(−0.13)

*, ** *Correlation coefficient significant at P 0.05 and P 0.01, respectively; The values inside the parentheses are for postrainy season, whereas the values outside the parentheses are for rainy season*.

### Combining ability analysis

Mean sum of squares for general combining ability of all the traits studied in the rainy season and postrainy seasons were significant at *P* = 0.01 (Table 6). Mean sum of squares due to SCA was significant for all the traits studied, during the rainy and postrainy seasons, except grain luster during rainy season and agronomic score and waxy bloom in the postrainy season indicating the role of both additive and non-additive nature of gene action in controlling most of the morphological and agronomic traits. The mean sum of squares due to reciprocal crosses was significant for days to 50% flowering and 100 seed weight across seasons, inflorescence exsertion during the rainy season; and plant height, panicle compactness, and panicle shape during the postrainy season, suggesting the influence of cytoplasmic factors in the expression of these traits.

**Table 6:** **Analysis of variance (ANOVA) showing mean sum of squares of general, specific and reciprocal combining abilities of F_1_(10 X 10) diallel across seasons (ICRISAT, Patancheru, 2013–14)**.

**Source**	**GCA**		**SCA**		**Reciprocal**		**Error**	
	**2013 R**	**2013 PR**	**2013 R**	**2013 PR**	**2013 R**	**2013 PR**	**2013 R**	**2013 PR**
Days to 50% flowering	75.23^**^	168.15^**^	10.31^**^	8.40^**^	2.74^**^	4.92^**^	1.27	1.96
Plant height (cm)	14639.30^**^	5747.19^**^	1856.50^**^	494.31^**^	40.27	69.04^**^	38.62	33.99
100 seed weight (g)	0.81^**^	2.07^**^	0.10^**^	0.18^**^	0.02^*^	0.05^**^	0.02	0.02
Grain yield (t/ha)	11.80^**^	44.52^**^	3.94^**^	12.95^**^	0.70	1.08	0.50	1.08
Agronomic score	6.83^**^	3.06^**^	0.44^**^	0.15	0.17	0.11	0.12	0.16
Inflorescence exsertion	2.14^**^	10.80^**^	0.34^**^	0.58^**^	0.11^**^	0.11	0.06	0.09
Panicle compactness	1.68^**^	1.74^**^	0.08^**^	0.17^**^	0.01	0.03^**^	0.01	0.01
Panicle shape	–	4.88^**^	–	1.40^**^	–	0.32^**^	–	0.13
Glume coverage	17.63^**^	21.08^**^	0.70^**^	0.49^**^	0.21	0.03	0.19	0.11
Awns	1.91^**^	1.85^**^	0.13^**^	0.13^**^	0.00	0.00	0.00	0.00
Grain luster	0.02^**^	0.04^**^	0.02	0.02^**^	0.00	0.01	0.00	0.00

*, ** *F probability significant at P 0.05 and P 0.01, respectively; GCA, general combining ability; SCA, specific combining ability; R, rainy season; PR, postrainy season*.

### Estimates of general combining ability (*gca*), specific combining ability (*sca*) and reciprocal effects

#### *gca* effects of agronomic traits

*gca* effects of days to 50% flowering ranged from −2.87 (Phule Anuradha) to 3.36 (ICSV 700) in the rainy season, and from −3.65 (CSV 15) to 4.40 (ICSV 700) in the postrainy season (Table 7). Phule Anuradha (−2.87^**^), ICSV 25019 (−1.85^**^), IS 2146 (−0.77^**^) and Swarna (−1.37^**^) exhibited significant negative *gca* effects in the rainy season, and Phule Anuradha (−1.61^**^), CSV 15 (−3.65^**^), ICSV 25019 (−2.58^**^), PS 35805 (−2.58^**^), and Swarna (−2.60^**^) exhibited significant negative *gca* effects for days to 50% flowering in the postrainy season. ICSV 700 (3.36^**^, 4.40^**^, respectively, in the rainy and postrainy season), M 35-1 (0.50^*^, 2.30^**^), IS 2123 (1.33^**^, 1.70^**^), and IS 18551 (2.46^**^, 3.17^**^) across seasons and IS 2146 (1.44^**^) in the postrainy season showed significant positive *gca* effects for days to 50% flowering.

**Table 7:** **Estimates of general combining ability of agronomic and panicle traits of parents (10 X 10 diallel) across seasons (ICRISAT, Patancheru, 2013–14)**.

**Traits**	**ICSV 700**	**Phule Anuradha**	**M 35-1**	**CSV 15**	**ICSV 25019**	**PS 35805**	**IS 2123**	**IS 2146**	**IS 18551**	**Swarna**
Days to 50% flowering	3.36^**^ (4.40^**^)	−2.87^**^ (−1.61^**^)	0.50^*^ (2.30^**^)	−0.35 (−3.65^**^)	−1.85^**^ (−2.58^**^)	−0.44 (−2.58^**^)	1.33^**^ (1.70^**^)	−0.77^**^ (1.44^**^)	2.46^**^ (3.17^**^)	−1.37^**^ (−2.60^**^)
Plant height (cm)	32.23^**^ (19.42^**^)	5.59^**^ (7.92^**^)	19.43^**^ (10.53^**^)	5.19^**^ (1.87)	−44.49^**^ (−28.69^**^)	−42.96^**^ (−27.69^**^)	3.87^**^ (−2.13)	5.96^**^ (1.53)	31.29^**^ (20.59^**^)	−16.10^**^ (−3.36^**^)
100 seed weight (g)	−0.02 (0.07^*^)	0.20^**^ (0.56^**^)	0.01 (0.33^**^)	0.09^**^ (0.01)	0.04 (−0.22^**^)	−0.07^**^ (−0.43^**^)	−0.06^*^ (−0.16^**^)	−0.26^**^ (−0.05)	−0.31^**^ (−0.40^**^)	0.38^**^ (0.29^**^)
Grain yield (t/ha)	0.23 (0.44^*^)	−0.43^**^ (0.52^*^)	−0.34^*^ (1.27^**^)	0.90^**^ (0.86^**^)	0.40^**^ (0.07)	0.48^**^ (0.03)	−0.14 (0.89^**^)	−1.79^**^ (−3.86^**^)	−0.06 (0.77^**^)	0.74^**^ (−0.99^**^)
Agronomic score	0.13 (0.04)	0.18^*^ (0.11)	0.26^**^ (0.11)	−0.46^**^ (−0.31^**^)	−0.73^**^ (−0.39^**^)	−0.75^**^ (−0.31^**^)	0.96^**^ (0.59^**^)	0.69^**^ (0.66^**^)	0.18^*^ (−0.01)	−0.47^**^ (−0.48^**^)
Inflorescence exsertion	−0.05 (−0.18^**^)	0.12^*^ (0.17^**^)	−0.13^*^ (−0.16*)	−0.23^**^ (−0.53^**^)	0.00 (−0.16*)	0.13^*^ (−0.21^**^)	0.57^**^ (1.51^**^)	0.35^**^ (0.96^**^)	−0.12^*^ (−0.34^**^)	−0.63^**^ (−1.06^**^)
Panicle compactness	−0.17^**^ (0.13^**^)	−0.13^**^ (0.00)	−0.15^**^ (0.13^**^)	−0.20^**^ (−0.50^**^)	−0.03 (−0.10^**^)	0.02 (0.05^*^)	0.62^**^ (0.37^**^)	0.43^**^ (0.37^**^)	−0.13^**^ (0.02)	−0.25^**^ (−0.47^**^)
Panicle shape	(0.41^**^)	(0.01)	(0.35^**^)	(−0.70^**^)	(−0.24^**^)	(0.20^**^)	(0.31^**^)	(0.58^**^)	(0.01)	(−0.94^**^)
Glume coverage	0.35^**^ (0.03)	−0.28^**^ (−0.31^**^)	−0.05 (−0.27^**^)	−0.65^**^ (−0.31^**^)	−0.65^**^ (−0.51^**^)	−0.55^**^ (−0.44^**^)	−0.01 (−0.37^**^)	−0.31^**^ (−0.41^**^)	2.52^**^ (2.89^**^)	−0.38^**^ (−0.31^**^)
Awns	0.24^**^ (0.25^**^)	0.24^**^ (0.21^**^)	0.24^**^ (0.23^**^)	−0.35^**^ (−0.35^**^)	−0.36^**^ (−0.35^**^)	−0.36^**^ (−0.35^**^)	0.24^**^ (0.25^**^)	0.24^**^ (0.23^**^)	0.25^**^ (0.25^**^)	−0.36^**^ (−0.35^**^)
Grain luster	(0.01)	(0.01)	(0.01)	(0.01)	(0.02)	(0.01)	(0.02)	(0.02)	(−0.13^**^)	(0.02)

*, ** *t-test significant at 0.05 and 0.01 probability levels; The values outside the parentheses are for rainy season and inside the parentheses are for postrainy season*.

*gca* effects for plant height ranged from −44.49 (ICSV 25019) to 32.23 (ICSV 700) in the rainy season, −28.69 (ICSV 25019) to 20.59 (IS 18551) in the postrainy season. ICSV 25019 (−44.49^**^ and −28.69^**^, respectively, in the rainy and postrainy seasons), PS 35805 (−42.96^**^ and −27.69^**^) and Swarna (−16.10^**^ and −3.36^**^) exhibited significant negative *gca* effects for plant height across seasons. ICSV 700, Phule Anuradha, M 35-1, CSV 15, IS 2123, IS 2146, and IS 18551 in the rainy season, and ICSV 700, Phule Anuradha, M 35-1, and IS 18551 in the postrainy season exhibited significant positive *gca* effects for plant height.

*gca* effects for 100 seed weight ranged from −0.31 (IS 18551) to 0.38 (Swarna) in the rainy and −0.43 (PS 35805) to 0.56 (Phule Anuradha) in the postrainy seasons. PS 35805 (−0.07^**^), IS 2123 (−0.06^**^), IS 2146 (−0.26^**^), and IS 18551 (−0.31^**^) in the rainy season, and ICSV 25019 (−0.22^**^), PS 35805 (−0.43^**^), IS 2123 (−0.16^**^), and IS 18551 (−0.40^**^) in the postrainy season exhibited significant negative *gca* effects for 100 seed weight. Whereas, the genotypes Phule Anuradha (0.20^**^), CSV 15 (0.09^**^), and Swarna (0.38^**^) in the rainy season, and ICSV 700 (0.07^*^), Phule Anuradha (0.56^**^), M 35-1 (0.33^**^), and Swarna (0.29^**^) in the postrainy season showed positive *gca* effects for 100 seed weight.

*gca* effects of sorghum grain yield ranged from −1.79 (IS 2146) to 0.90 (CSV 15) in the rainy season, and −3.86 (IS 2146) to 1.27 (M 35-1) in the postrainy season. The *gca* effects of Phule Anuradha (−0.43^**^), M 35-1 (−0.34^*^), and IS 2146 (−1.79^**^) in the rainy season, and IS 2146 (−3.86^**^) and Swarna (−0.99^**^) in the postrainy season exhibited significant negative *gca* effects for grain yield. The genotypes CSV 15 (0.90^**^), ICSV 25019 (0.40^**^), PS 35805 (0.48^**^), and Swarna (0.74^**^) in the rainy season and ICSV 700 (0.44^*^), Phule Anuradha (0.52^*^), M 35-1 (1.27^**^), CSV 15 (0.86^**^), IS 2123 (0.89^**^), and IS 18551 (0.77^**^) in the postrainy season showed significant positive *gca* effects for grain yield. *gca* effects of agronomic score ranged from −0.75 (PS 35805) to 0.96 (IS 2123) in the rainy season, and −0.48 (Swarna) to 0.66 (IS 2146) in the postrainy season.

#### *gca* effects of morphological traits

The *gca* effects of inflorescence exsertion ranged from −0.63 (Swarna) to 0.57 (IS 2123) in the rainy season, and −1.06 (Swarna) to 1.51 (IS 2123) in the postrainy season (Table 7). *gca* effects of panicle compactness ranged from −0.25 (Swarna) to 0.62 (IS 2123) in the rainy season, and −0.50 (CSV 15) to 0.37 (IS 2123 and IS 2146) in the postrainy season. Six genotypes exhibited significant and negative *gca* effects and two genotypes exhibited positive and significant *gca* effects for panicle compactness in the rainy season. Three genotypes exhibited significant negative *gca* effects, while five genotypes exhibited significant positive *gca* effects for panicle compactness in the postrainy season.

*gca* effects of the panicle shape ranged from −0.94 (Swarna) to 0.58 (IS 2146) in the postrainy season. The general combining ability of glume cover ranged from −0.65 to 2.52 in the rainy season, and −0.51 to 2.89 in the postrainy season. All the genotypes exhibited significant negative *gca* effects except IS 18551 (2.52^**^ and 2.89^**^) with significant positive *gca* effects for glume coverage across seasons. *gca* effects of awns ranged from −0.36 to 0.25 in the rainy season, and −0.35 to 0.25 in the postrainy season. CSV 15, ICSV 25019, PS 35805, and Swarna exhibited significant negative *gca* effects, while ICSV 700, Phule Anuradha, M 35-1, IS 2123, IS 2146, and IS 18551 exhibited significant positive *gca* effects for presence of awns across seasons.

### Specific combining ability (*sca*) effects

#### *sca* effects of agronomic traits

The *sca* effects for days to 50% flowering ranged from −3.66 to 3.19 and −3.20 to 3.61 during the rainy and postrainy season, respectively. For plant height the *sca* effects ranged from −71.09 to 45.68 and −30.09 to 22.75, for 100 seed weight from −0.56 to 0.37 and −0.50 to 0.38, for grain yield from −1.24 to 2.65 and −3.24 to 4.70, respectively, in the rainy and postrainy seasons. For agronomic score from −1.02 to 0.91 in the rainy season (Table 8). Ten hybrids in the rainy season and nine hybrids in the postrainy season exhibited significant negative *sca* effects for days to 50% flowering. ICSV 700 X CSV 15 across seasons, and Phule Anuradha X PS 35805, M 35-1 X CSV 15 in the postrainy season showed significant positive *sca* effects for days to 50% flowering.

**Table 8:** **Estimates of specific combining ability effects of agronomic traits of F_1_ crosses (10 X 10 diallel) of sorghum across seasons (ICRISAT, Patancheru, 2013–14)**.

**Pedigree**	**Days to 50% flowering**		**Plant height (cm)**		**100 seed weight (g)**		**Grain yield (t/ha)**		**Agronomic score**
	**2013 R**	**2013 PR**	**2013 R**	**2013 PR**	**2013 R**	**2013 PR**	**2013 R**	**2013 PR**	**2013 R**
ICSV 700 X Phule Anuradha	−0.80	1.30	−8.32^*^	6.19	−0.06	−0.05	−0.11	1.22	0.00
ICSV 700 X M 35-1	−3.66^**^	−2.62^**^	−18.26^**^	−2.53	0.08	0.37^**^	1.16^*^	−0.03	−0.25
ICSV 700 X CSV 15	3.19^**^	3.16^**^	7.10	−4.98	−0.23^**^	−0.18	0.05	2.90^**^	−1.02^**^
ICSV 700 X ICSV 25019	−0.15	0.10	36.21^**^	16.69^**^	0.09	0.32^**^	0.21	1.91^**^	−0.09
ICSV 700 X PS 35805	−0.06	−0.74	34.11^**^	21.24^**^	0.01	0.04	1.51^**^	1.33	−0.07
ICSV 700 X IS 2123	−2.83^**^	−2.02^^**^^	−13.80^**^	5.13	0.04	0.14	0.29	1.21	0.05
ICSV 700 X IS 2146	−0.90	0.25	−4.79	−3.53	0.25^**^	−0.06	−0.13	−1.55^^**^^	−0.34
ICSV 700 X IS 18551	−2.80^**^	−1.99^^**^^	−7.89	−9.81^^**^^	0.02	0.20^^**^^	0.83	−0.36	−0.16
ICSV 700 X Swarna	−0.46	−0.05	16.72^**^	12.47^**^	0.05	0.38^**^	−0.07	−0.34	0.82^**^
Phule Anuradha X M 35-1	0.57	−0.77	−32.19^**^	−2.14	−0.21^**^	−0.04	−0.24	−0.77	−0.46^**^
Phule Anuradha X CSV 15	0.09	−0.99	9.85^**^	9.30^**^	0.18^**^	0.13	−1.21^**^	−1.60^**^	0.26
Phule Anuradha X ICSV 25019	−0.41	−1.72	34.53^**^	14.86^**^	0.27^**^	0.32^**^	0.22	−0.11	0.20
Phule Anuradha X PS 35805	−0.16	3.61^**^	35.76^**^	22.75^**^	0.24^**^	−0.12	0.92^**^	4.70^**^	0.21
Phule Anuradha X IS 2123	−0.60	0.16	−11.09^**^	−6.70	−0.14	−0.06	0.12	0.19	−0.16
Phule Anuradha X IS 2146	0.50	0.10	−7.02	−11.48^**^	−0.21^**^	−0.04	0.79	−0.15	−0.22
Phule Anuradha X IS 18551	−1.06	−1.80	−13.49^**^	−11.09^**^	−0.19^**^	−0.10	1.18^**^	1.99^**^	−0.55^**^
Phule Anuradha X Swarna	−0.06	−1.20	29.75^**^	6.19	0.21^**^	0.23^**^	0.36	−0.52	0.10
M 35-1 X CSV 15	−2.95^**^	2.76^**^	−0.64	8.35^**^	0.25^**^	0.03	−0.31	0.35	0.18
M 35-1 X ICSV 25019	−0.45	0.70	45.68^**^	21.69^**^	0.21^**^	0.18	0.28	2.82^**^	0.28
M 35-1 X PS 35805	−2.20^**^	−0.97	35.01^**^	10.13^**^	0.23^**^	0.20^**^	0.04	1.03	0.46^**^
M 35-1 X IS 2123	−0.63	−0.09	−4.89	−2.09	−0.06	−0.05	−0.23	1.55^**^	−0.41
M 35-1 X IS 2146	−0.70	−0.15	−17.54^**^	−4.65	−0.13	0.15	0.41	−0.72	0.20
M 35-1 X IS 18551	0.74	−0.39	−15.10^**^	−5.92	0.05	−0.11	1.64^**^	1.60^**^	−0.63^**^
M 35-1 X Swarna	1.07	−0.29	26.18^**^	2.47	−0.22^**^	−0.20^**^	−0.04	0.27	−0.15
CSV 15 X ICSV 25019	−0.60	−1.19	−16.73^**^	−10.76^**^	−0.24^**^	0.08	1.83^**^	1.16	−0.66^**^
CSV 15 X PS 35805	−0.35	−1.69	−3.80	−10.09^**^	−0.15	0.15	1.50^**^	0.38	−0.06
CSV 15 X IS 2123	−1.61^**^	−0.64	−0.65	−0.64	0.14	−0.02	2.65^**^	1.71^**^	0.48^**^
CSV 15 X IS 2146	−1.01	−2.70^**^	1.70	−2.09	0.34^**^	−0.01	−0.13	−0.32	−0.08
CSV 15 X IS 18551	−1.91^**^	−1.27	15.80^**^	7.75^**^	0.10	−0.02	1.02^**^	1.31	−0.24
CSV 15 X Swarna	−0.41	−0.84	28.76^**^	17.80^**^	−0.12	0.21^**^	−1.06^**^	1.04	0.91^**^
ICSV 25019 X PS 35805	2.49^**^	0.75	−71.09^**^	−30.09^**^	−0.56^**^	−0.49^**^	−1.24^**^	−3.24^**^	−0.21
ICSV 25019 X IS 2123	−0.11	−0.70	18.73^**^	3.80	0.26^**^	0.34^**^	0.69	2.37^**^	0.58^**^
ICSV 25019 X IS 2146	−0.01	−0.44	24.70^**^	5.69	0.23^**^	0.45^**^	−0.51	0.31	0.77^**^
ICSV 25019 X IS 18551	−1.08	−1.84^**^	31.60^**^	20.52^**^	0.06	0.17	−0.75	2.05^**^	0.03
ICSV 25019 X Swarna	−1.91^**^	0.43	−38.22^**^	−17.76^**^	0.06	−0.50^**^	2.29^**^	0.29	−0.66^**^
PS 35805 X IS 2123	−0.36	−3.20^**^	26.92^**^	5.02	0.37^**^	0.22*	−0.17	1.19	0.43
PS 35805 X IS 2146	−1.60^**^	−1.94^**^	22.06^**^	9.69^**^	0.22^**^	0.36^**^	−0.65	0.69	0.20
PS 35805 X IS 18551	−1.16	−2.34^**^	38.97^**^	20.63^**^	0.13	0.13	−0.31	0.99	−0.12
PS 35805 X Swarna	0.00	1.26	−38.93^**^	−15.98^**^	−0.24^**^	−0.33^**^	1.73^**^	0.29	−0.64^**^
IS 2123 X IS 2146	1.30	1.78	0.80	6.91	−0.33^**^	0.02	0.02	−0.90	−0.67^**^
IS 2123 X IS 18551	0.74	1.21	−25.08^**^	−2.15	−0.10	−0.16	−0.65	−1.25	0.17
IS 2123 X Swarna	−0.43	0.48	18.95^**^	1.80	0.15	0.15	0.32	0.50	0.15
IS 2146 X IS 18551	0.00	1.65	−17.72^**^	−9.14^**^	−0.03	0.05	0.07	−0.26	0.28
IS 2146 X Swarna	−1.00	−3.09^**^	16.32^**^	21.47^**^	−0.08	0.06	−0.27	0.07	−0.07
IS 18551 X Swarna	−0.73	0.35	27.64^**^	17.97^**^	0.22^**^	0.18	0.40	1.68^**^	0.27

*, ** *t-test significant at P 0.05 and P 0.01 respectively; R, rainy season; PR, postrainy season*.

Significant negative *sca* effects for plant height were observed for 14 hybrids in the rainy season, and 10 hybrids in the postrainy season. Fifteen hybrids across seasons, Phule Anuradha X Swarna, ICSV 25019 X IS 2123, ICSV 25019 X IS 2146, PS 35805 X IS 2123, IS 2123 X Swarna in the rainy season, and M 35-1 X CSV 15 in the postrainy season exhibited significant positive *sca* effects for plant height.

Significant negative *sca* effects for grain yield were observed in Phule Anuradha X CSV 15, ICSV 25019 X PS 35805 across seasons, CSV 15 X Swarna in the rainy season, and ICSV 700 X IS 2146 in the postrainy season. Four hybrids across seasons, seven in the rainy season and eight hybrids in the postrainy season, exhibited significant and positive *sca* effects for grain yield. ICSV 700 X CSV 15, Phule Anuradha X M 35-1, Phule Anuradha X IS 18551, M 35-1 X IS 18551, CSV 15 X ICSV 25019, ICSV 25019 X Swarna, PS 35805 X Swarna, IS 2123 X IS 2146 exhibited significant negative *sca* effects while ICSV 700 X Swarna, M 35-1 X PS 35805, CSV 15 X IS 2123, CSV 15 X Swarna, ICSV 25019 X IS 2123, ICSV 25019 X IS 2146 exhibited significant positive *sca* effects for agronomic score in the rainy season.

#### *sca* effects of morphological traits

The *sca* effects of the inflorescence exsertion ranged from −0.92 to 0.95 and −0.91 to 1.11, for panicle compactness from −0.52 to 0.37 and −0.67 to 0.50, for glume coverage −1.22 to 0.78 and −1.09 to 0.71, for awns from −0.25 to 0.36 and 0.25 to 0.35, respectively, in the rainy and postrainy seasons. For panicle shape it ranged from −1.46 to 1.60 and grain luster from −0.16 to −0.01 in the postrainy season (Table 9). The genotypes with significant *gca* and/or *sca* for morphological traits can be utilized in developing sorghum cultivars for use by the farmers.

**Table 9:** **Estimates of specific combining ability effects of panicle traits of F_1_ crosses (10 X 10 diallel) of sorghum, across seasons (ICRISAT, Patancheru, 2013–14)**.

**Pedigree**	**Inflorescence exsertion**		**Panicle compactness**		**Panicle shape**	**Glume coverage**		**Awns**		**Grain luster**
	**2013 R**	**2013 PR**	**2013 R**	**2013 PR**	**2013 PR**	**2013 R**	**2013 PR**	**2013 R**	**2013 PR**	**2013 PR**
ICSV 700 X Phule Anuradha	0.02	0.43^*^	−0.03	0.23^**^	0.82^**^	−0.42	−0.43^*^	0.16^**^	0.19^**^	0.01
ICSV 700 X M 35-1	0.10	0.43^*^	−0.02	0.10	0.49^*^	−0.65^*^	−0.46^*^	0.16^**^	0.17^**^	0.01
ICSV 700 X CSV 15	0.03	−0.37	0.03	−0.10	−0.63^**^	−0.39	−0.43^*^	−0.25^**^	−0.25^**^	0.01
ICSV 700 X ICSV 25019	−0.37^*^	−0.41^*^	−0.13	−0.17^*^	−0.43	−0.39	−0.23	−0.24^**^	−0.25^**^	−0.01
ICSV 700 X PS 35805	−0.17	−0.86^**^	−0.18^*^	0.18^*^	0.64^**^	−0.49	−0.29	−0.24^**^	−0.25^**^	0.01
ICSV 700 X IS 2123	−0.10	0.26	0.22^**^	−0.13	−0.48^*^	−0.35	−0.36	0.16^**^	0.15^**^	−0.01
ICSV 700 X IS 2146	−0.05	0.14	0.07	−0.13	−0.41	−0.05	−0.33	0.16^**^	0.17^**^	−0.01
ICSV 700 X IS 18551	−0.08	0.61^**^	−0.03	0.22^**^	0.82^**^	0.78^**^	−0.63^**^	0.15^**^	0.15^**^	−0.02
ICSV 700 X Swarna	0.43^**^	−0.17	0.08	−0.30^**^	−1.23^**^	−0.32	−0.09	−0.24^**^	−0.25^**^	−0.01
Phule Anuradha X M 35-1	0.27	0.08	−0.05	−0.27^**^	−0.61^**^	−0.687 ^*^	−0.13	0.16^**^	0.04	−0.16^**^
Phule Anuradha X CSV 15	−0.30	−0.56^**^	0.00	−0.13	−0.56^*^	−0.09	−0.09	−0.25^**^	−0.21^**^	0.01
Phule Anuradha X ICSV 25019	−0.37^*^	−0.59^**^	−0.17^*^	−0.37^**^	−1.03^**^	0.25	0.11	−0.24^**^	−0.21^**^	−0.01
Phule Anuradha X PS 35805	−0.33^*^	0.13	−0.05	−0.02	0.04	−0.19	0.04	−0.24^**^	−0.21^**^	0.01
Phule Anuradha X IS 2123	0.07	−0.09	0.18^*^	0.00	−0.08	−0.05	−0.03	0.16^**^	0.19^**^	−0.01
Phule Anuradha X IS 2146	0.28	0.46^*^	0.20^*^	0.00	−0.35	0.25	0.01	0.16^**^	0.04	−0.01
Phule Anuradha X IS 18551	0.25	0.26	−0.07	0.35^**^	1.22^**^	−0.59^*^	0.71^**^	0.15^**^	0.19^**^	0.14^**^
Phule Anuradha X Swarna	−0.07	−0.36	0.05	−0.17^*^	−0.66^**^	−0.02	−0.09	−0.24^**^	−0.21^**^	−0.01
M 35-1 X CSV 15	−0.38^*^	−0.56^**^	0.02	−0.10	−0.40	−0.32	0.54^*^	−0.25^**^	−0.23^**^	0.01
M 35-1 X ICSV 25019	−0.62^**^	−0.76^**^	−0.15	−0.33^**^	−0.86^**^	0.01	0.07	−0.24^**^	−0.23^**^	−0.01
M 35-1 X PS 35805	−0.92^**^	−0.54^**^	−0.03	0.18^*^	0.70^**^	0.58^*^	0.01	−0.24^**^	−0.23^**^	0.01
M 35-1 X IS 2123	0.15	0.24	0.03	−0.13	−0.41	−0.29	−0.06	0.16^**^	0.17^**^	−0.01
M 35-1 X IS 2146	0.37^*^	0.79^**^	0.22^**^	−0.13	−0.68^**^	−0.65^*^	−0.03	0.16^**^	0.19^**^	−0.01
M 35-1 X IS 18551	0.17	0.43^*^	−0.05	0.22^**^	0.89^**^	0.51	0.34	0.15^**^	0.17^**^	0.14^**^
M 35-1 X Swarna	0.52^**^	−0.02	0.07	0.37^**^	0.34	0.41	−0.13	−0.24^**^	−0.23^**^	−0.01
CSV 15 X ICSV 25019	0.48^**^	1.11^**^	−0.10	−0.03	−0.31	0.28	0.11	0.35^**^	0.35^**^	−0.01
CSV 15 X PS 35805	0.52^**^	0.33	−0.15	−0.18^*^	−0.75^**^	0.18	0.04	0.35^**^	0.35^**^	0.01
CSV 15 X IS 2123	0.08	0.61^**^	0.08	0.50^**^	0.80^**^	0.31	−0.03	−0.25^**^	−0.25^**^	−0.01
CSV 15 X IS 2146	−0.53^**^	−0.01	−0.07	0.50^**^	1.20^**^	−0.05	0.34	−0.25^**^	−0.23^**^	−0.01
CSV 15 X IS 18551	−0.40^*^	−0.37	0.00	−0.15^*^	−0.89^**^	−0.55	−0.29	−0.10^**^	−0.25^**^	−0.02
CSV 15 X Swarna	−0.05	0.18	0.12	−0.67^**^	0.89^**^	0.35	−0.09	0.35^**^	0.35^**^	−0.01
ICSV 25019 X PS 35805	0.95^**^	0.96^**^	−0.15	0.42^**^	1.29^**^	0.18	0.24	0.36^**^	0.35^**^	−0.01
ICSV 25019 X IS 2123	0.02	0.08	0.08	0.10	0.17	−0.02	0.17	−0.24^**^	−0.25^**^	−0.02
ICSV 25019 X IS 2146	0.07	0.63^**^	0.10	0.10	0.57^*^	−0.05	0.21	−0.24^**^	−0.23^**^	−0.02
ICSV 25019 X IS 18551	−0.63^**^	−0.91^**^	−0.17^*^	−0.22^**^	−0.53^*^	−0.55	−1.09^**^	−0.25^**^	−0.25^**^	0.13^**^
ICSV 25019 X Swarna	−0.28	−0.02	−0.05	−0.07	−0.58^*^	0.01	0.11	0.36^**^	0.35^**^	−0.02
PS 35805 X IS 2123	−0.12	0.13	0.03	−0.05	−0.26	0.21	0.11	−0.24^**^	−0.25^**^	−0.01
PS 35805 X IS 2146	0.10	0.34	0.22^**^	−0.05	−0.03	−0.15	0.14	−0.24^**^	−0.23^**^	−0.01
PS 35805 X IS 18551	−0.43^**^	−0.36	−0.22^**^	−0.53^**^	−1.46^**^	−0.32	−0.49^*^	−0.25^**^	−0.25^**^	−0.02
PS 35805 X Swarna	−0.47^*^	−0.14	−0.10	−0.22^**^	−1.01^**^	−0.09	0.04	0.36^**^	0.35^**^	−0.01
IS 2123 X IS 2146	0.17	−0.87^**^	−0.38^**^	−0.37^**^	−0.65^**^	−0.02	0.07	0.16^**^	0.17^**^	−0.02
IS 2123 X IS 18551	0.47^**^	0.43^*^	0.18^*^	−0.02	−0.08	−0.19	0.11	0.15^**^	0.15^**^	0.13^**^
IS 2123 X Swarna	−0.02	0.64^**^	0.13	0.47^**^	1.37^**^	−0.62^*^	−0.03	−0.24^**^	−0.25^**^	−0.02
IS 2146 X IS 18551	0.52^**^	−0.36	0.37^**^	−0.02	−0.35	−1.22^**^	−0.53^*^	0.15^**^	0.17^**^	0.13^**^
IS 2146 X Swarna	−0.30	−0.81^**^	−0.52^**^	0.47^**^	1.60^**^	0.35	0.01	−0.24^**^	−0.23^**^	−0.02
IS 18551 X Swarna	−0.17	−0.01	0.05	−0.18^*^	−0.83^**^	0.18	0.37	−0.25^**^	−0.25^**^	0.13^**^

*, ** *Significant at P 0.05 and P 0.01 probability levels; R, rainy season; PR, postrainy season*.

### Reciprocal combining ability effects of agronomic and morphological traits

M 35-1 X ICSV 700, ICSV 25019 X M 35-1, IS 18551 X ICSV 700, IS 18551 X M 35-1, IS 18551 X PS 35805, IS 18551 X IS 2123 in the rainy season, PS 35805 X Phule Anuradha in the postrainy season, and PS 35805 X Phule Anuradha across seasons, exhibited significant negative reciprocal effects for days to 50% flowering (Table 10). CSV 15 X ICSV 700 and Swarna X IS 18551 in the rainy season, six hybrids in the postrainy season, and Swarna X M 35-1 and Swarna X IS 2123 across seasons exhibited significant positive reciprocal effects for days to 50% flowering. The crosses CSV 15 X Phule Anuradha, PS 35805 X Phule Anuradha, PS 35805 X ICSV 700, and Swarna X CSV 15 exhibited significant negative and the crosses Phule Anuradha X ICSV 700, ICSV 25019 X M 35-1, IS 18551 X ICSV 700, and Swarna X IS 2123 showed significant positive reciprocal effects for plant height in the postrainy season.

**Table 10:** **Estimates of reciprocal combining ability effects of reciprocal crosses (10 X 10 diallel) of sorghum across seasons (ICRISAT, Patancheru, 2013–14)**.

**Pedigree**	**Days to 50% flowering**		**Plant height (cm)**	**100 seed weight (g)**		**Inflorescence exsertion**	**Panicle compactness**	**Panicle shape**
	**2013 R**	**2013 PR**	**2013 PR**	**2013 R**	**2013 PR**	**2013 R**	**2013 PR**	**2013 PR**
Phule Anuradha X ICSV 700	−0.50	5.33^**^	11.11^**^	0.03	−0.20*	–	–	–
M 35-1 X ICSV 700	−1.70^*^	0.33	−0.56	−0.02	−0.15	0.17	–	–
M 35-1 X Phule Anuradha	−0.33	0.83	−5.00	0.02	0.13	−0.17	0.17^*^	0.50^*^
CSV 15 X ICSV 700	3.00^**^	1.50	1.67	−0.05	0.02	–	−0.17^*^	−0.50^*^
CSV 15 X Phule Anuradha	−1.00	0.33	−7.78^*^	−0.05	−0.02	0.17	–	−0.17
CSV 15 X M 35-1	0.67	3.00^**^	−0.56	0.03	−0.38^**^	−0.17	0.17^*^	0.67^**^
ICSV 25019 X ICSV 700	−0.83	0.50	0.56	0.12	0.08	0.50^**^	−0.17^*^	−0.50^*^
ICSV 25019 X Phule Anuradha	−0.33	0.33	−1.67	−0.20^**^	−0.08	0.30^*^	−0.17^*^	−0.50^*^
ICSV 25019 X M 35-1	−1.70^*^	−3.00^**^	8.89^*^	−0.07	0.17	−0.17	−0.33^**^	−1.00^**^
ICSV 25019 X CSV 15	−0.67	−1.50	−4.44	–	0.25^*^	0.17	–	−0.17
PS 35805 X ICSV 700	−2.00^**^	0.67	0.56	−0.03	–	0.17	–	–
PS 35805 X Phule Anuradha	−0.67	−3.67^**^	−17.22^**^	0.02	0.13	0.17	−0.33^**^	−1.00^**^
PS 35805 X M 35-1	−0.67	−0.33	−12.78^**^	−0.02	−0.05	–	–	–
PS 35805 X CSV 15	0.33	–	2.78	0.05	0.22^*^	–	–	−0.17
PS 35805 X ICSV 25019	−0.33	−0.50	–	−0.02	0.18	–	–	–
IS 2123 X ICSV 700	–	−0.67	5.56	−0.03	−0.03	−0.30^*^	–	–
IS 2123 X Phule Anuradha	−0.67	−1.17	−2.22	0.02	0.10	–	–	–
IS 2123 X M 35-1	−1.33	0.83	0.56	0.10	–	−0.50^**^	–	–
IS 2123 X CSV 15	0.50	−1.33	−5.56	−0.08	0.02	−0.30^*^	–	0.17
IS 2123 X ICSV 25019	−1.17	−0.67	2.78	0.05	0.15	0.50^**^	–	–
IS 2123 X PS 35805	–	1.83^*^	−5.00	−0.08	−0.12	−0.17	–	–
IS 2146 X ICSV 700	1.17	–	−0.56	−0.02	0.18	−0.17	–	–
IS 2146 X Phule Anuradha	1.00	0.83	−2.22	−0.12	0.13	–	–	–
IS 2146 X M 35-1	−1.17	0.50	−0.56	−	0.12	−0.17	–	–
IS 2146 X CSV 15	–	3.00^**^	4.45	0.02	−0.03	0.50^**^	–	−0.17
IS 2146 X ICSV 25019	−0.17	–	3.89	0.20^**^	0.13	–	–	–
IS 2146 X PS 35805	−0.67	−1.17	5.56	0.07	−0.07	−0.17	–	0.17
IS 2146 X IS 2123	–	0.83	−1.67	0.03	−0.10		–	–
IS 18551 X ICSV 700	−1.80^*^	−0.50	10.00^*^	0.13	0.05	−0.30^*^	–	–
IS 18551 X Phule Anuradha	–	–	−0.56	−0.02	0.02	−0.17	–	–
IS 18551 X M 35-1	−2.50^**^	–	−5.00	0.03	−0.03	−0.17	–	–
IS 18551 X CSV 15	−1.00	−0.17	−6.67	–	–	0.17	–	−0.17
IS 18551 X ICSV 25019	−0.33	1.00	5.56	–	−0.03	−0.17	0.33^**^	1.00^**^
IS 18551 X PS 35805	−1.70^*^	−0.17	−2.22	0.10	−0.08	−0.17	−0.17^*^	−0.50^*^
IS 18551 X IS 2123	−2.00^**^	2.00^*^	−0.56	−0.12	−0.17	−0.17	–	–
IS 18551 X IS 2146	−0.50	0.50	7.22	0.12	0.05	−0.30^*^	–	–
Swarna X ICSV 700	−0.33	0.67	−6.11	0.08	−0.08	0.30^*^	–	–
Swarna X Phule Anuradha	0.83	1.83^*^	−2.78	0.07	−0.13	0.30^*^	–	−0.17
Swarna X M 35-1	2.30^**^	2.33^*^	7.22	−0.40^**^	−0.67^**^	0.30^*^	0.33^**^	1.50^**^
Swarna X CSV 15	0.67	0.83	−9.45^*^	0.20^*^	−0.22^*^	–	–	–
Swarna X ICSV 25019	−0.33	1.17	−1.11	0.20^**^	0.05	–	–	–
Swarna X PS 35805	−0.33	−0.33	−5.00	0.08	−0.12	–	–	–
Swarna X IS 2123	1.70^*^	2.17^*^	10.56^**^	0.15	−0.07	0.17	–	0.17
Swarna X IS 2146	0.33	1.00	−0.55	0.02	0.18	−0.30^*^	–	–
Swarna X IS 18551	1.80^*^	−1.17	−3.89	0.03	0.05	–	–	–

*, ** *t-test significant at 0.05 and 0.01 probability levels; ORS, overall resistance score; R, rainy season; PR, postrainy season*.

ICSV 25019 X Phule Anuradha in the rainy season and Phule Anuradha X ICSV 700, CSV 15 X M 35-1, Swarna X CSV 15 in the postrainy season, and Swarna X M 35-1 across seasons, exhibited significant negative reciprocal effects for 100 seed weight. IS 2146 X ICSV 25019, Swarna X CSV 15, Swarna X ICSV 25019 in the rainy season and ICSV 25019 X CSV 15, PS 35805 X CSV 15 in the postrainy season exhibited significant positive reciprocal effects for 100 seed weight.

### Combining ability estimates and genetic parameters

Variance due to GCA (σ^2^ g) was higher than the variance due to SCA (σ^2^ s) for glume cover across seasons (Table 11); agronomic score and waxy bloom in the rainy season and days to 50% flowering and inflorescence exsertion in the postrainy season also showed high *gca* variance, indicating the predominance of additive gene action in controlling the expression of these traits. Plant height and grain yield exhibited higher σ^2^ s than the σ^2^ g across seasons; days to 50% flowering and inflorescence exsertion in the rainy season, and panicle shape in the postrainy season showed high σ^2^ s than the variance due to *gca*, indicating the predominance of non-additive type of gene action in controlling the expression of these traits. The other traits that had similar σ^2^ g and σ^2^ s exhibited both additive and non-additive type of gene action.

**Table 11:** **Estimates of various genetic parameters for different agronomic and panicle traits of sorghum across seasons (ICRISAT, Patancheru, 2013–14)**.

**Traits**	**Days to 50% flowering**	**Plant height (cm)**	**100 seed weight (g)**	**Grain yield (t/ha)**	**Agronomic score**	**Inflorescence exsertion**	**Panicle compactness**	**Panicle shape**	**Glume coverage**	**Awns**	**Grain lustre**
σ^2^g	3.70	730.03	0.04	0.57	0.34	0.10	0.08	(0.24)	0.87	0.10	–
	(8.31)	(285.66)	(0.10)	(2.17)	(0.15)	(0.54)	(0.09)		0.87	0.10	
σ^2^s	9.05	1817.89	0.08	3.44	0.32	0.27	0.07	(1.27)	0.51	0.12	0.02
	(6.44)	(460.32)	(0.16)	(11.87)		(0.49)	(0.16)		(0.38)	(0.12)	(0.01)
σ^2^r	0.74 (1.48)	(17.53)	(0.02)	–	–	0.03	(0.01)	(0.1)	–	–	–
σ^2^e	1.27	38.62	0.02	0.50	0.12	0.06	0.01	(0.13)	0.19	–	–
	(1.96)	(33.99)	(0.02)	(1.08)	(0.16)	(0.09)	(0.01)		(0.11)		
σ^2^a	7.40	1460.07	0.08	1.13	0.67	0.21	0.17	(0.48)	1.74	0.19	–
	(16.62)	(571.32)	(0.20)	(4.34)	(0.29)	(1.07)	(0.17)		(2.10)	(0.19)	
σ^2^d	9.05	1817.89	0.08	3.44	0.32	0.27	0.07	(1.27)	0.51	0.12	0.02
	(6.44)	(460.32)	(0.16)	(11.87)		(0.49)	(0.16)		(0.38)	(0.12)	(0.01)
σ^2^p	18.45	3317.39	0.18	5.17	1.14	0.57	0.25	(1.97)	2.46	0.32	0.02
	(26.50)	(1083.15)	(0.40)	(17.29)	(0.41)	(1.66)	(0.35)		(2.55)	(0.31)	(0.02)
h${}_{\text{n}{\text{s}}^{2}}$	0.40	0.44	0.45	0.22	0.59	0.37	0.68	(0.24)	0.71	0.60	0.10
	(0.63)	(0.53)	(0.51)	(0.25)	(0.71)	(0.65)	(0.50)		(0.82)	(0.60)	(0.17)
h${}_{{\text{b}}^{2}}$	0.89	0.99	0.90	0.88	0.87	0.85	0.95	(0.89)	0.92	1.00	1.00
	(0.87)	(0.95)	(0.91)	(0.94)	(0.67)	(0.94)	(0.94)		(0.97)	(0.99)	(0.78)
GCA/SCA ratio	0.41	0.40	0.49	0.16	1.04	0.38	1.26	(0.19)	1.70	0.77	0.06
	(1.29)	(0.62)	(0.64)	(0.18)		(1.09)	(0.55)		(2.73)	(0.75)	(0.14)
Predictability ratio	0.45	0.45	0.5	0.25	0.68	0.43	0.72	(0.27)	0.77	0.61	0.10
	(0.72)	(0.55)	(0.56)	(0.27)		(0.69)	(0.53)		(0.85)	(0.6)	(0.22)

*σ^2^g, gca variance; σ^2^s, sca variance; σ^2^r, reciprocal variance; σ^2^e, environmental/error variance; σ^2^a, additive variance; σ^2^d, dominance variance; σ^2^p, phenotypic variance; h${}_{\text{n}{\text{s}}^{\text{2}}}$, narrow–sense heritability; h${}_{{\text{b}}^{2}}$, broad–sense heritability; GCA, general combining ability; SCA, specific combining ability; The values in the parentheses are for postrainy season and off the parentheses are for rainy season*.

Glume cover showed greater additive (σ^2^ a) than the dominance variance (σ^2^ d) across seasons. Agronomic score, waxy bloom, and panicle compactness in the rainy season, and days to 50% flowering, plant height, 100 seed weight, and inflorescence exsertion in the postrainy season showed higher additive variance than the dominance variance. Overall resistance score, grain yield, and plant color exhibited higher dominance variance than the additive variance across seasons. Days to 50% flowering, plant height, and inflorescence exsertion in the rainy season and panicle shape in the postrainy season possessed higher dominance variance than the additive variance.

Glume cover and awns exhibited high GCA/SCA ratios across seasons. Agronomic score, waxy bloom, and panicle compactness in the rainy season, and days to 50% flowering, plant height, 100 seed weight, and inflorescence exsertion in the postrainy season exhibited high GCA/SCA ratios, indicating the additive type of gene action controlling the expression of these traits. Panicle compactness, glume cover, and awns showed high predictability ratios across seasons. The predictability ratios for agronomic score, and waxy bloom in the rainy season, and days to 50% flowering, plant height, 100 seed weight, and inflorescence exsertion in the postrainy season were quite high. Heritability estimates of the traits studied ranged from 0.10 to 0.71 (narrow-sense heritability), and 0.85 to 1.00 (broad-sense heritability) in the rainy season, and 0.17 to 0.82 (narrow-sense heritability), and 0.67 to 0.99 (broad-sense heritability) in the postrainy season. Almost all the traits exhibited moderate to high heritability values, except grain yield and grain luster across the seasons. Panicle shape in the postrainy season exhibited low (≤ 0.30) narrow-sense heritability.

## Discussion

Significance of *F*-values for all the traits studied indicated the presence of high variability in the parents used for developing the full diallel. Plant height, 100 seed weight and grain yield were associated with susceptibility to shoot fly. Days to 50% flowering, agronomic score, inflorescence exsertion, panicle compactness, panicle shape, glume coverage, and awns were associated with shoot fly resistance.

Association between the shoot fly resistance, morphological, and agronomic traits suggested complex interactions between shoot fly and plant traits. Significance of the GCA and SCA mean sum of squares for all the traits across seasons suggested that both the additive and non-additive nature of gene action for agronomic and panicle characteristics. The significance of reciprocal combining ability effects for days to 50% flowering, plant height, and 100 seed weight, suggesting possible role of cytoplasmic factors in inheritance of these traits. These interactions should be taken into consideration while developing strategies for improving sorghums for shoot fly resistance and high grain yield.

Genotypes with negative GCA effects for days to 50% flowering can be utilized to develop the hybrids with early flowering. To develop hybrids with moderate height, care should be taken to select the parents with moderate plant height. Additive gene action in the rainy season and dominance in the postrainy season for days to 50% flowering and plant height suggested G X E interactions for these traits. This contrasting gene expression in the rainy and postrainy seasons for days to 50% flowering and plant height suggested the season specific breeding of these traits for sorghum improvement. Meng et al. (1998), Rafiq et al. (2002), and Mohammed Maarouf (2009) reported additive gene action for days to 50% flowering, while Erenso (1998) reported additive gene action for plant height. Higher magnitude of SCA variance was reported by Manickam and Vijendra Das (1994) and Umakanth et al. (2002) for both the plant traits. High GCA/SCA and predictability ratios for 100 seed weight in the postrainy season indicated the predominance of additive gene action, whereas both additive and non-additive gene action was observed in the rainy season. Grain yield exhibited higher SCA variance suggesting the predominance of dominance (non-additive) type of gene action (Wilson et al., 1978; Singhania, 1980; Amsalu, 1987; Hovny et al., 2000; Umakanth et al., 2002; Girma et al., 2010). However, the importance of both the additive and non-additive gene action was observed for 100 seed weight by Toure et al. (1996).

Knowledge of the genetic architecture of grain yield, and morphological and agronomic traits will be useful for formulating a meaningful breeding strategy for developing improved genotypes. Genetic diversification of sorghum for key traits is important for sustaining the yield gains and mapping QTL underlying agronomically important traits is a key step in understanding their genetic control and for using the tightly linked markers for marker-assisted breeding for crop improvement (Srinivas et al., 2009; Ashok Kumar et al., 2011; Nagaraja Reddy et al., 2013, 2014). Many studies were conducted in identifying the QTL regions of different traits associated with insect resistance as well as the morphological and agronomic traits (Satish et al., 2009; Srinivas et al., 2009; Aruna et al., 2011b; Nagaraja Reddy et al., 2013, 2014). Based on the present inheritance studies of the agronomic and morphological traits and as well as the QTL information available one can effectively plan suitable breeding strategies for sorghum improvement.

Most of the hybrids studied exhibited higher grain yield than the parents even if one of the parent was high yielding, suggesting that one of the parent should possess high grain yield ability for developing high yielding hybrids. This is very critical in sorghum improvement considering the fact that hybrids are preferred over the varieties worldover, barring Africa. Most of the commercial hybrids show 30–40% yield superiority over the best varieties in a given ecology. The panicle trait such as inflorescence exsertion exhibited predominance of additive gene action in the postrainy season, and dominance gene action in the rainy season, while glume cover and presence of awns showed predominance of additive gene action.

The genotypes CSV 15, ICSV 25019, PS 35805, and Swarna exhibited negative *gca* effects for almost all the traits, but positive *gca* effects for grain yield. Hence, these genotypes can be effectively used in breeding the high yielding sorghums. The crosses involving the genotype IS 2146 either as male or female parent showed a decrease in grain yield being a poor combiner coupled with low *per se* mean yield. Phule Anuradha and M 35-1 showed positive *gca* effects for 100 seed weight but negative *gca* effects for grain yield in the rainy season, and positive effects in the postrainy season, suggesting that these genotypes can be effectively utilized for breeding high yielding shoot fly resistant sorghums for the postrainy season. Both M 35-1 and Phule Anuradha are highly adapted to postrainy environments, and are very popular with farmers. ICSV 25019, PS 35805, IS 2123, and IS 18551 exhibited negative *gca* effects for 100 seed weight, but showed positive *gca* effects for grain yield. Hence, these genotypes can be utilized for breeding high yielding sorghums with shoot fly resistance. Though the genotypes CSV 15 and Swarna showed positive *gca* effects for 100 seed weight and grain yield, but these may not be good parents in a shoot fly resistance breeding program. ICSV 700, IS 2123, and IS 18551 showed positive *gca* effects for most of the traits and these can be utilized for improving shoot fly resistance. Higher narrow- and broad-sense heritability estimates suggested high heritability of these traits across environments, and greater role of additive gene action, suggesting that selection is effective for improving these traits. This information can be used for developing high yielding cultivars with insect resistance for sustainable crop production.

## Conclusions

Genotypic response varies across seasons, and hence, it is important to identify genotypes with desirable agronomic traits, insect resistance, and high grain yield for different seasons and locations. The genotypes ICSV 700, Phule Anuradha, M 35-1, ICSV 25019, PS 35805, IS 2123, and IS 18551 exhibiting moderate to high shoot fly resistance and desirable agronomic traits, can be effectively used in sorghum improvement. Both additive and non-additive type of gene action governs the morphological (inflorescence exsertion, panicle compactness, and panicle shape) and agronomic (days to 50% flowering, plant height and 100 seed weight) traits and hence it is important to exploit heterosis breeding for improving agronomic and morphological traits and grain yield in sorghum. The significance of reciprocal effects for some of the traits (days to 50% flowering, plant height, and 100 seed weight) suggested that apart from the direct genetic effects, the cytoplasmic factors also played an important role in inheritance of these traits. An understanding of the association between shoot fly resistance and morphological and agronomic traits will be useful to improve the strategies to develop shoot fly-resistant cultivars with desirable plant types, and season specific adaptation for sustainable crop production.

### Conflict of interest statement

The authors declare that the research was conducted in the absence of any commercial or financial relationships that could be construed as a potential conflict of interest.
