# Supplementary material for: Quantitative genetic analysis of agronomic and morphological traits in sorghum, Sorghum bicolor
Source: Front Plant Sci. 2015 Nov 3;6:945. doi: 10.3389/fpls.2015.00945 (PMC4630571; doi:10.3389/fpls.2015.00945)
Supplement: Supplementary file 1 [file DataSheet1.DOCX]

| **Annexure I. Pedigrees of the sorghum genotypes used in crossing program for diallel analysis (ICRISAT, Patancheru, 2013-14).** | | | |
| --- | --- | --- | --- |
| **S. no.** | **Genotype** | **Pedigree** |  |
| 1 | ICSV 700 | (IS 1082 x SC 108-3)-1-1-1-1-1 |  |
| 2 | Phule Anuradha | RSLG 559 x RSLG 1175 |  |
| 3 | M 35-1 | Selection from local land race Maldandi |  |
| 4 | CSV 15 | (ICSV 112 x SPV 462) |  |
| 5 | ICSV 25019 | (ICSV 705 x YT-1-69)-10-1-1-2-1 |  |
| 6 | PS 35805 | (((IS 5622 x CS 3541)-6-1-1-1-1) x (SC 108-3 x CS 3541)-19-1)-1-2-1-1 |  |
| 7 | IS 2123 | PI 195683 |  |
| 8 | IS 2146 | Kaura, PI 221569 (Landrace, Nigeria) |  |
| 9 | IS 18551 | Jijwejere 935 (Landrace, Ethiopia) |  |
| 10 | Swarna | IS 18463 |  |
